# The podocytes’ inflammatory responses in experimental GN are independent of canonical MYD88-dependent toll-like receptor signaling

**DOI:** 10.1038/s41598-024-52565-8

**Published:** 2024-01-27

**Authors:** Thomas Schömig, Paul Diefenhardt, Ingo Plagmann, Bastian Trinsch, Tim Merz, Giuliano Crispatzu, David Unnersjö-Jess, Jasper Nies, David Pütz, Claudio Sierra Gonzalez, Bernhard Schermer, Thomas Benzing, Paul Thomas Brinkkoetter, Sebastian Brähler

**Affiliations:** 1 Department II of Internal Medicine and Center for Molecular Medicine Cologne, University of Cologne and University Hospital Cologne, Cologne, Germany; 2 Cluster of Excellence Cellular Stress Response in Aging-associated Diseases (CECAD), Faculty of Medicine and University Hospital Cologne, University of Cologne, Cologne, Germany

**Keywords:** Glomerular diseases, Toll-like receptors, Podocytes

## Abstract

Podocytes form the kidney filtration barrier and continuously adjust to external stimuli to preserve their integrity even in the presence of inflammation. It was suggested that canonical toll-like receptor signaling, mediated by the adaptor protein MYD88, plays a crucial role in initiating inflammatory responses in glomerulonephritis (GN). We explored the influence of podocyte-intrinsic MYD88 by challenging wild-type (WT) and podocyte-specific *Myd88* knockout (MyD88^pko^) mice, with a model of experimental GN (nephrotoxic nephritis, NTN). Next-generation sequencing revealed a robust upregulation of inflammatory pathways and changes in cytoskeletal and cell adhesion proteins in sorted podocytes from WT mice during disease. Unchallenged MyD88^pko^ mice were healthy and showed no proteinuria, normal kidney function and lacked morphological changes. During NTN, MyD88^pko^ exhibited a transient increase in proteinuria in comparison to littermates, while histological damage, podocyte ultrastructure in STED imaging and frequencies of infiltrating immune cells by flow cytometry were unchanged. MYD88-deficiency led to subtle changes in the podocyte transcriptome, without a significant impact on the overall podocyte response to inflammation, presumably through MYD88-independent signaling pathways. In conclusion, our study reveals a comprehensive analysis of podocyte adaptation to an inflammatory environment on the transcriptome level, while MYD88-deficiency had only limited impact on the course of GN suggesting additional signaling through MYD88-independent signaling.

## Introduction

Podocytes are highly specialized epithelial cells that constitute the core of the renal filtration barrier. Their limited capacity for regeneration underscores the importance of preserving their structure and function. Any impairment or loss of podocytes can result in reduced renal function, proteinuria, and eventually, end-stage kidney disease. In certain conditions like minimal change disease and membranous nephropathy, podocytes are directly damaged^1^. In contrast, rapid and progressive glomerulonephritis (RPGN) primarily targets endothelial cells (e.g., anti-neutrophil cytoplasmic antibody (ANCA)-associated GN) or the glomerular basement membrane (anti-GBM-GN)^2,3^. However, the subsequent infiltration of immune cells and production of proinflammatory cytokines during RPGN create a highly inflammatory environment, which eventually harms podocytes^4^.

We have recently demonstrated that a podocyte-specific knockout of the NF-κB-essential modulator (NEMO), a central molecule of the pro-inflammatory NF-κB signaling cascade, can reduce proteinuria in a model of experimental GN (nephrotoxic nephritis, NTN) by decreasing the secretion of inflammatory cytokines^5^. Moreover we found that signaling through NEMO causes alterations of the actin cytoskeleton through small GTPases of the Rho family^6^. Despite the association between most glomerular diseases and a pro-inflammatory environment, the mechanisms by which podocytes detect and respond to these stimuli are not fully understood. Toll-like receptors (TLRs) are among the most prominent receptor families responsible for transmitting inflammatory signals to cells, comprising 10 members in humans and 13 members in rodents^7^. These signaling pathways can be divided into two distinct classes based on their downstream adaptor proteins.

The TIR domain-containing adaptor-inducing IFNβ (TRIF)-dependent pathway, which is activated by TLR3 and TLR4, is primarily active in macrophages and dendritic cells to create type 1 interferon responses^8,9^. TLR1,2,5–9 signaling, on the other hand, leads to the activation of myeloid differentiation factor 88 (MYD88) dependent pathways. MYD88, a cytoplasmic adaptor protein, is not only central in TLR signal transduction, but is also involved in IL-1R signaling, acting via IRAK1, IRAK4, IRF7, TRAF6 and NEMO to ultimately activate NF-κB-dependent gene products^7,10^. As a result, TLR stimulation induces the transcription of pro-inflammatory cytokines such as IFNα, IL-6, IL-1β, TNFα as well as chemokines such as IP-10 and MIG^10,11^.

Within the kidney, TLRs are found in various cell types, including podocytes, and can be triggered by different stimuli like lipid A and fibrinogen^12^. Furthermore, in murine models of glomerular inflammation such as cryoglobulinemic MPGN and streptozotocin-induced diabetic nephropathy, an increase in TLR expression within the kidney is observed as the disease progresses. Depleting TLR2 and TLR4 has been shown to reduce renal inflammation in these models^13–15^. Notably, studies in a murine model of membranoproliferative GN revealed a connection between disease severity and the expression of TLR9 in the periglomerular area^16^. In humans, TLR2 and TLR4 are predominantly found in endothelial cells and podocytes, whereas TLR9 is almost exclusively localized in podocytes. Patients with ANCA-associated GN exhibited a significant upregulation of these TLRs. Intriguingly, the expression levels of TLR2 and TLR4 correlate with both the extent of histological damage and decline in renal function, suggesting that TLR signaling plays a central role in human disease^17^.

TLR signaling through MYD88 is known for its central role in initiating the NF-κB pathway and activating the innate immune response. However, there is a lack of experimental evidence regarding the functional role of TLR signaling in podocytes. In this study, we aimed to investigate the transcriptomic response of podocytes during glomerular inflammation and to assess the influence of MYD88-dependent signaling in podocytes under both healthy and diseased conditions.

## Materials and methods

### Animals

*Myd88*^flox^ mice were a kind gift from Manolis Pasparakis^18^. They were mated with our recently published *Nphs2*.2a.iCre.2a.mTomato (Pod^TOM^) mouse, which combines podocyte-specific Cre-expression with expression of the fluorophore mTomato^19^. Mice were kept on a C57BL/6N background and housed under specific pathogen-free conditions. Animal experiments were performed according to national and institutional animal care and ethical guidelines (Animal welfare office, University of Cologne, Germany) and were approved by the State office of North Rhine-Westphalia, Germany, Department of Nature, Environment and Consumer protection (LANUV NRW, approval AZ 81-02.04.2019.A085).

### Animal experiments and functional studies

Groups of Pod^TOM^ x *Myd88*^*flox/flox*^ mice (referred to as MyD88^pko^) and Pod^wt/wt^ x *Myd88*^*flox/flox*^ littermate controls (referred to as WT) were analyzed between the ages of 8–52 weeks. NTN was induced in 8- to 16-weeks old, male MyD88^pko^ and WT controls by tail vein injection of 9 µl per gram body weight of nephrotoxic sheep serum (PTX-001 Sheep Anti-GBM, Probetex Inc., Texas, USA) on two consecutive days. The dosage of this batch of nephrotoxic serum was based on a recent publication^20^. Organs were harvested at the time points indicated in the figures and in the figure legends after cardial perfusion with cold PBS. For fluorescent activated cell sorting (FACS), MyD88^pko^ mice and Pod^TOM^ controls were used. All methods are reported in accordance with the ARRIVE guidelines.

Albuminuria was determined in spot urine by standard ELISA (Bethyl Laboratories). Urinary creatinine was measured using a standard assay (Cayman Chemicals). BUN was measured using standard laboratory methods or colorimetric assay (Cayman Chemicals).

### Morphometric studies

For histology, 2 µm sections of FFPE kidney tissues were stained with periodic-acid-Schiff and acid Fuchsin Orange G following standard protocols. For immunohistochemistry, 4 µm thick sections were blocked with peroxidase blocking buffer and Avidin/Biotin Blocking Kit (SP-2001, Vector Laboratories), stained with antibodies directed against WT1 (abcam, clone CAN-R9(IHC)-56-2, diluted 1:1000), and developed using an HRP immunodetection Kit (Vectastain Elite ABC anti-mouse Kit) and DAB staining solution (DAB Substrate Kit, Vector Laboratories). 25 glomerular cross-sections per kidney section were analyzed in a blinded fashion, and the positive cells were counted manually. For IF, mouse kidneys were frozen, and embedded in Tissue-Tek OCT (Sakura). Sections were fixed with 4% PFA and blocked using 5% normal donkey serum with 0.1% Triton-sodium azide. Slides were incubated with antibodies against Podocin (P0372, Sigma-Aldrich) and Cy3 coupled donkey anti-sheep-IgG (Jackson ImmunoResearch), followed by AF488 coupled donkey anti-rabbit antibody for 1 h at room temperature (Jackson Immuno Research) and mounted with ProLong Gold Antifade Reagent with DAPI (Invitrogen). IF was analyzed using a Zeiss Apotome 2 microscope.

### Stimulation emission depletion (STED) microscopy and semi-automated quantification of slit diaphragm length

The protocol was performed as previously described^21^. Briefly, formalin-fixed kidney tissue was incubated in hydrogel solution (4% vol/vol acrylamide, 0.25% wt/vol VA-044 initiator, and PBS) and cut using a Vibratome. Sections were incubated in clearing solution (200 mM boric acid and 4% SDS, pH 8.5) for 16 h at 50 °C and thereafter washed in 0.1% Triton X in PBS (PBST). Cleared sections were incubated with primary antibodies at 37 °C for 24 h followed by secondary antibodies (24 h at 37 °C). A rabbit anti-Podocin primary antibody (catalog no. P0372; RRID: AB_261982; 1:100; Sigma-Aldrich) and a donkey anti-rabbit Abberior STAR 635P secondary antibody (1:50) were used to stain for Podocin. The secondary antibodies were conjugated as follows: fluorophores (Atto-594 NHS ester; catalog no. 08741; Sigma-Aldrich or Abberior STAR 635P NHS ester; catalog no. 07679; Sigma-Aldrich) were conjugated to a donkey anti-rabbit IgG (catalog no. A16037; RRID: AB_2534711; Thermo Fisher Scientific, Bremen, Germany), 1 M NaHCO_3_ was added at a dilution of 1:10 to ensure basic conjugation conditions. The fluorophores were dissolved in DMSO at a concentration of 10 mg/ml. Fluorophores and antibodies were mixed at a 20-fold molar excess of fluorophores and incubated on a shaker (1 h at room temperature). A centrifugal filter (Amicon Ultra 0.5 centrifugal filter 30-MW cutoff; catalog no. UFC5030; Sigma-Aldrich) and centrifugation at 14,000×*g* (10 min) was used to remove the excess fluorophores. After filling up with PBS containing 0.1% sodium azide, the centrifugation step was repeated, and PBS containing 0.1% sodium azide was added for a final antibody concentration of 1 mg/ml. Morphometric analyses of the STED images and semi-automated detection and quantification were performed with ImageJ/Fiji software using a recently published algorithm^21,22^.

### Renal leukocyte isolation

Kidneys were minced and incubated in digestion medium (RPMI 1640 medium containing 10% FCS, 1% Penicillin/Streptomycin, 250 µg/ml Collagenase B, and 30U/ml DNase) at 37 °C for 45 min, then dissociated using the gentleMACS dissociator (Miltenyi Biotec). Percoll gradient (37% Percoll; GE Healthcare, Chalfont St. Giles, Great Britain) centrifugation was performed. The leukocyte layer was carefully aspirated and transferred to a 96-well plate or flow cytometry tubes for antibody staining.

### Staining for flow cytometry

Cells were pre-incubated with anti-CD16/32 antibody (Biolegend) for 5 min at 4 °C. Fluorochrome-labeled antibodies were added and incubated for 30 min at 4 °C. The following antibodies were used for stainings: anti-CD45 (30-F11), anti-CD19 (1D3) purchased from BD Bioscience, anti-CD4 (GK1.5), anti-CD8a (53–6.7), anti-TCRb (H57-597), anti-CD11b (M1/70), anti-F4/80 (BM8), anti-NK1.1 (PK136), anti-Ly6C (HK1.4) and anti-Ly6G (1A8) all purchased from Biolegend. Zombie Aqua staining (Biolegend) was used to exclude dead cells during flow cytometry. Data was acquired on a BD LSR Fortessa Cytometer (BD Bioscience).

## Podocyte isolation and RNA isolation

Podocytes were isolated from the kidneys as previously described^23^. Kidneys were harvested, and both renal arteries were dissected. Kidneys were perfused with 2 ml of Dynabeads solution (Dynabeads M-450 tosylactivated, Thermo Fisher Scientific) through the renal artery. After mincing, an enzymatic digestion was performed with Collagenase II (300U/ml, Worthington), Pronase E (1 mg/ml, Sigma-Aldrich and DNAse I (50 U/µl, Applichem) at 37 °C for 15 min. After sieving through a 100 µm strainer, glomeruli were isolated using a DynaMag magnet (DynaMag-2, Invitrogen). Glomeruli were then disrupted through a combination of enzymatic digestion and manual disruption by repetitive pipetting for 45 min at 37 °C. After sieving through a 40 µm cell strainer, cells were stained with DAPI and sorted using a BD FACSAria III (BD Bioscience). Living podocytes were defined as DAPI^neg^mTomato^+^. Flow cytometry of isolated glomeruli revealed 85.67% living cells, of which 19.88% were podocytes. RNA was isolated following manufacturers protocol (RNAqueous Micro-Kit, Thermo Fisher Scientific). Extraction quality control was performed using the Agilent 2200 TapeStation.

### Pre-amplification, library prep and RNA sequencing

Due to the low amount of input material, pre-amplification using the Ovation RNASeq System V2 was performed. cDNA synthesis, library preparation and quantification was performed as was described previously^24^. Sequencing was performed on a NovaSeq 6000 sequencing instrument with a 2 × 100 bp paired-end sequencing setup.

### Bulk RNA-sequencing analysis

After demultiplexing, adapters were trimmed using cutadapt 3.5. Reads were then pseudoaligned to the mm10 / GRCm38 reference genome using kallisto-0.43.1^25^. Differential isoform expression analysis was conducted using sleuth-0.3.0 (R-3.5.0)^26^. Ensembl v90 was used to annotate transcripts, while pathway analysis was facilitated using ConsensusPathDB-r35 with default settings (Pathway-based sets; minimum overlap with input list: 2; p-value cutoff: 0.01). Unannotated genes were removed from further analysis.

### Quantitative PCR for isolated podocytes and renal cortex

Total RNA was extracted from isolated podocytes as described above and from renal cortex using Direct-zol RNA Miniprep kit (R2050, Zymo Research, Irvine, USA), and then reverse transcribed into cDNA using High-Capacity cDNA Reverse Transcription Kit (Thermo Fisher Scientific). Quantitiative PCR was performed on an Applied Biosystems 7500 Fast Real-Time PCR System with standard protocol and following TaqMan gene expression assays (Thermo Fisher Scientific): *Myd88* (Mm00440338_m1), *Ccl2* (Mm00441242_m1), *Il1b* (Mm00434228_m1) and *Actb* (Mm02619580_g1) as the internal control for normalization. Data were analyzed using the ΔΔCt method.

## Results

### Bulk RNA sequencing of sorted podocytes reveals profound transcriptional changes in cytoskeletal, adhesion and proinflammatory pathways during GN

**Figure 1:**
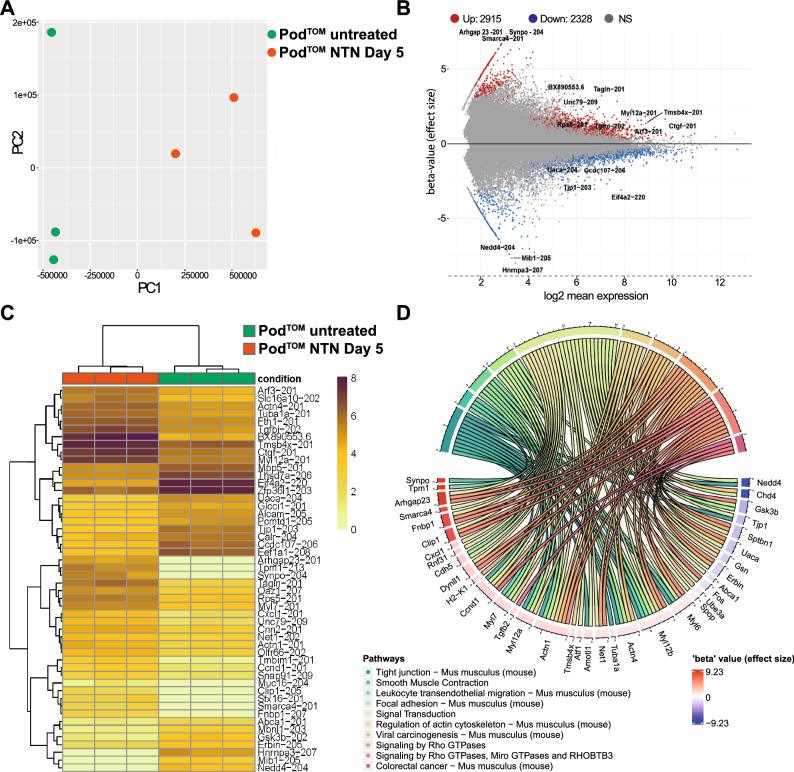
Bulk RNA sequencing of sorted podocytes under healthy conditions (Pod^TOM^ untreated) and on day 5 after NTN induction (Pod^TOM^ NTN Day 5) reveals profound transcriptional changes in cytoskeletal, adhesion and proinflammatory pathways during GN. **(A)** Principal component analysis of podocytes from healthy and nephritic mice reveals robust clustering into healthy (n = 3) and injured conditions (n = 3). **(B)** MA-Plot of differentially and significantly expressed genes (adj. p-value < 0.05) reveals 2915 up- and 2328 downregulated genes in Pod^TOM^ NTN Day 5 vs. Pod^TOM^ untreated podocytes. If multiple transcripts were present, only the one with CCDS entry, high transcript support level and highest effect size (in that order) was considered. **(C)** Heatmap of the top 50 regulated genes with adj. p-value < 0.05. **(D)** Chord plot of the top 10 significantly enriched biological pathways (q-value < 0.1) linked among the 100 top-regulated genes ordered by their beta-value (effect size).

To gain a comprehensive insight into the transcriptional changes during experimental GN, bulk-RNA sequencing was performed on sorted podocytes under healthy conditions and on day 5 after NTN induction. FACS was facilitated by the expression of mTomato under the control of the podocin promoter^19^. Principal component analysis (PCA) confirmed the clustering of podocytes into their respective experimental group (Fig. 1A). A total of 2915 transcripts were upregulated, and 2382 were downregulated (Fig. 1B, C). We analyzed the top 100 most significantly up-and downregulated genes (sorted by ascending adjusted p-value) enriched in pathway-based GO:terms. This analysis revealed a pronounced upregulation of genes associated with the actin cytoskeleton and its regulation. Key genes, such as *Actn1, Actn4, Fnbp1* and *Arhgap23*, which are critical for actin cytoskeletal organization and remodeling through small Rho GTPases, along with genes pivotal for cell contractility like *Tpm1, Myl12b, Myl6* were significantly upregulated. This upregulation reflects the extensive structural remodeling observed in podocytes, known as foot process effacement (Fig. 1D). Notably, Synpo, an actin-associated protein, was the most differentially expressed gene, underscoring the recently described protective function in adriamycin-induced podocyte injury^27^. Among the most differentially expressed genes was *Cxcl1*, encoding a potent chemoattractant for neutrophil granulocytes as well as other chemokine genes including *Tgfb2*, *Cxcl10*, *Cxcl12* and *Cxcl15* (Fig. 5E). Among the TLR genes, *Tlr1*, *Tlr2*, and *Tlr4* were significantly upregulated whereas *Tlr7* was downregulated. *Tlr3*, which is associated with the MYD88-independent pathway, was not regulated.

### Maintenance of podocyte function and structure is independent of MYD88

**Figure 2:**
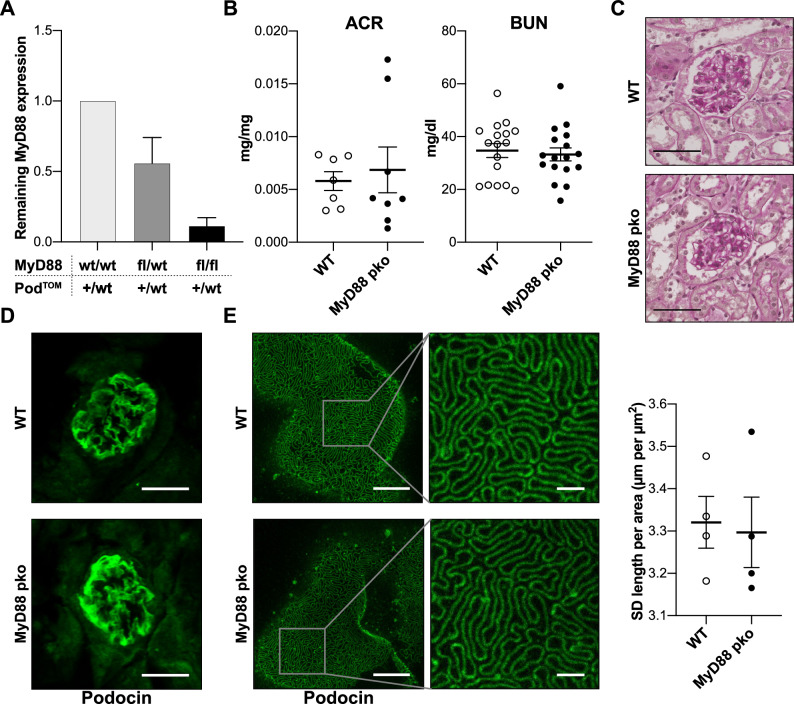
Maintenance of podocyte function and structure is independent of MYD88. **(A)** Real time qPCR for *Myd88* expression in podocytes isolated by FACS using the podocyte-specific mTomato fluorochrome of mice with heterozygous Podocin.2a.iCre.2a.mTomato (Pod^TOM^) background. Comparison between mice with following additional genotypes: *Myd88* wild type (wt/wt) (n = 3), heterozygous *Myd88* flox (fl/wt) (n = 3) and *Myd88* flox/flox (fl/fl, MyD88^pko^) (n = 3). The analysis reveals a sufficient podocyte-specific depletion of *Myd88* in MyD88^pko^ mice compared to MyD88^wt/wt^, and a partial *Myd88* knockdown in MyD88^fl/wt^ mice. (**B**) No significant difference in baseline kidney function is observed in albumin-to-creatinine ratio (ACR, n = 7 vs. 8) and blood urea nitrogen levels (BUN, n = 17 each) of 8–14 weeks old (n = 17 vs. 17), untreated male WT and MyD88^pko^ littermates. (**C**) Representative pictures of PAS stainings showing no glomerular abnormalities in untreated MyD88^pko^ and WT mice. Scale bars 30 µm. **(D)** Additionally, no difference is seen in baseline immunofluorescence staining for the slit diaphragm protein podocin (Scale bar: 30 µm). **(E)** Representative images of STED microscopy and quantification of slit diaphragm length of healthy MyD88^pko^ mice (n = 4) and wild type littermate controls (n = 4). Scale bar overview: 5 µm. Scale bar high magnification: 1 µm.

To investigate the role of MYD88-dependent TLR signaling in podocytes during renal homeostasis and glomerular inflammation, mice with a podocyte-specific knockout of *Myd88* were generated (Pod^TOM^ x *Myd88*^flox/flox^ = MyD88^pko^) and compared with healthy littermate controls (*Myd88*^flox/flox^ = WT). Successful knockout of *Myd88* in mice was confirmed by qPCR of sorted podocytes (Fig. 2A). MyD88^pko^ mice were observed up to 52 weeks of age and remained viable and fertile, showing no overt phenotype. Confirming these observations, no proteinuria or elevated blood urea nitrogen (BUN) levels were detected in mice up to 52 weeks of age (Fig. 2B and Supplemental Fig. 1). Morphologically, glomeruli appeared normal in PAS stainings and had no changes in the expression of the slit diaphragm protein podocin (Fig. 2C, D). As light microscopy is prone to miss subtle changes in podocyte architecture and slit diaphragm morphology, we employed stimulated emission depletion (STED) microscopy in combination with a recently published semi-automated quantification algorithm to quantify SD length and morphology, revealing no differences in MyD88^pko^ mice compared to age-matched wild type control mice (Fig. 2E)^21^.

### MYD88-deficiency in podocytes does not influence the course of experimental glomerulonephritis

**Figure 3:**
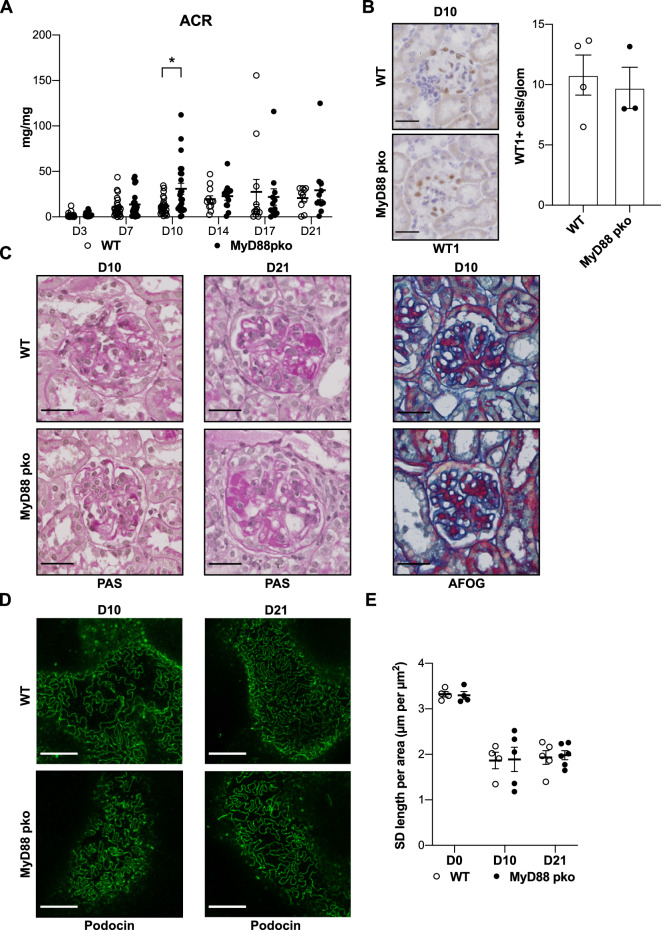
MYD88-deficiency in podocytes does not influence the course of experimental glomerulonephritis. (**A**) Albumin-to-creatinine ratio (ACR) shows a significant albeit transient increase of proteinuria in MyD88^pko^ mice 10 days after NTS injection (Day 3–10: n = 24 each. Day 14–21 n = 12 each). *p < 0.05, unpaired t-test. (**B**) Quantification of WT1 positive cells per glomerulus. Depicted is the mean with SD of WT (n = 4) vs MyD88^pko^ (n = 3) (**C**) Representative PAS stainings at day 10 and day 21 of NTN showing PAS-positive glomerular deposits in both groups. Additionally, AFOG staining reveals no difference in glomerular fibrosis at day 10 after injection. Scale bars: 30 µm. **(D)** Representative pictures of STED microscopy showing changed morphology of the slit diaphragm at day 10 and 21 of NTN with reduced slit diaphragm coverage of basal membrane. Scale bars: 5 µm. **(E)** Semi-automated quantification of the slit diaphragm coverage per area showing a significant reduction in coverage at indicated time points after NTN induction compared to untreated mice (D0), but no significant difference between MyD88^pko^ mice and wild type controls (D0: n = 4 each, D10: n = 4 vs. 5, D21: n = 5 vs. 6).

Having shown that MYD88 in podocytes is dispensable for renal homeostasis and the maintenance of podocyte function and structure, we next aimed to investigate its role in glomerular inflammation. To this end, NTN was induced in MyD88^pko^ mice and WT controls. IF staining for podocin and sheep IgG 10 days after disease induction confirmed equal glomerular binding of nephrotoxic sheep IgG in both control and MyD88^pko^ mice (Supplemental Fig. 2A). After NTN induction, MyD88^pko^ and littermates developed proteinuria, with a significant but only transient increase in proteinuria seen in MyD88^pko^ mice at day 10 (Fig. 3A). Both, control and MyD88^pko^ mice, showed similar numbers of WT1-positive cells at day 10 of NTN indicating no increased podocyte loss (Fig. 3B). In line, we observed similar extent of histological damage in PAS and AFOG stained kidney sections (Fig. 3C). STED microscopy revealed comparable shortening of the slit diaphragm in nephritic MyD88^pko^ and WT controls, indicating similar levels of foot process effacement. Of note, despite declining proteinuria on day 21 of NTN, the extent of morphological changes in the foot processes persisted at this late stage of disease (Fig. 3D, E and Supplemental Fig. 2B).

**Figure 4:**
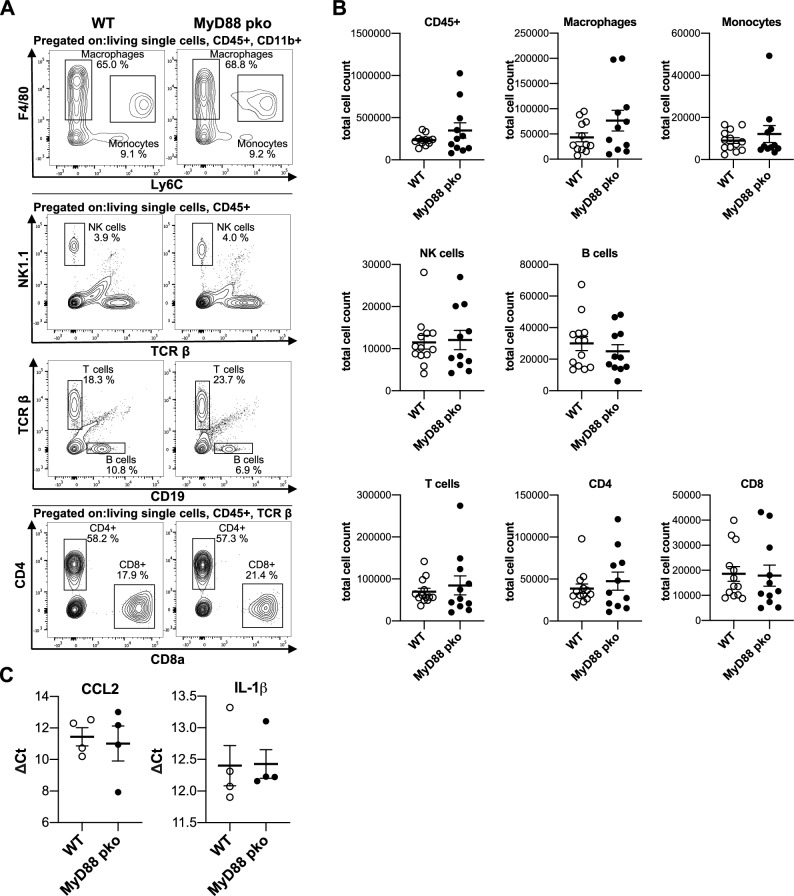
Absence of MYD88 signaling in podocytes has no impact on the composition of renal immune cells during NTN. **(A)** Gating strategy for flow cytometry analysis of indicated immune cell subsets in the kidney of MyD88^pko^ (n = 12) and WT mice at day 10 of NTN (n = 13). **(B)** Flow cytometry analysis shows no difference in leucocyte numbers (CD45^+^) at day 10 after induction, as well as no difference in the following immune cell subsets: macrophages (CD45^+^ CD11b^+^ F4/80^+^ Ly6C^-^), monocytes (CD45^+^ CD11b^+^ F4/80^INT^ Ly6C^+^), NK cells (CD45^+^ NK1.1^+^ TCRβ^-^), B cells (CD45^+^ CD19^+^ TCRβ^-^), T cells (CD45^+^ TCRβ^+^) and the T cell-subsets T helper cells (CD45^+^ TCRβ^+^ CD4^+^ CD8^-^) and cytotoxic T cells (CD45^+^ TCRβ^+^ CD8^+^ CD4^-^). (**C**) Real time qPCR for *Ccl2* and *Il-1β* normalized to *Actb* in kidney cortex reveals no difference for general pro-inflammatory signaling between MyD88^pko^ (n = 4) and controls (n = 4).

In line with the unchanged functional and histological parameters, no differences were observed in the number of renal macrophages, monocytes, NK cells, B cells, CD4^+^ T helper cells and CD8^+^ cytotoxic T cells (Fig. 4A, B). In addition, transcript levels cortical *Ccl2* and *Il1-β*, two proinflammatory signaling molecules, did not differ between the groups using qPCR (Fig. 4C).

### Bulk RNA sequencing of nephritic MyD88^pko^ podocytes reveals MYD88 independent signaling pathways in NTN

Considering the profound transcriptional changes in podocytes during NTN, including NF-κB dependent genes, the apparent absence of a renal phenotype in MyD88^pko^ mice pointed towards a potential compensatory mechanism. To pin down potential genes involved, we compared the transcriptome from nephritic mTomato-positive podocytes (Pod^TOM^) (one of the groups from Fig. 1) with the transcriptome of nephritic MyD88^pko^ mice.

**Figure 5:**
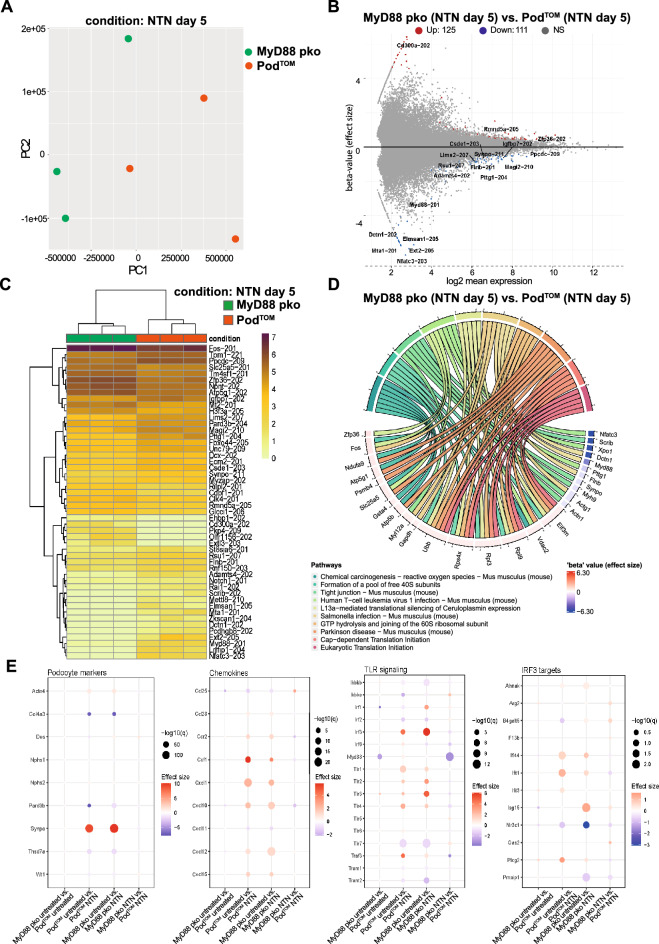
Bulk RNA sequencing of nephritic MyD88^pko^ podocytes reveals MyD88 independent signaling pathways in GN. **(A)** Principal component analysis of podocytes isolated from nephritic mice at day 5 after NTS injection with intact MyD88 signaling (Pod^TOM^) (n = 3) and nephritic mice with podocyte specific *Myd88* knock out (MyD88^pko^) (n = 3). **(B)** MA-Plot of differentially and significantly expressed genes (adj. p-value < 0.05) genes reveals 125 up- and 111 downregulated genes. If multiple isoforms were present, only the one with CCDS entry, high transcript support level and highest effect size (in that order) was considered. **(C)** Heat map of the top 50 regulated and significant genes (adj. p-value < 0.05). **(D)** Chord plot of top 10 significantly enriched pathways (q-value < 0.1) linked among the 100 top-regulated genes ordered by their beta-value (effect size). **(E)** Expression profiles of podocyte markers, cytokine receptors, chemokines/cytokines and members of the TLR signaling pathways for the indicated comparisons between groups (p-value < 0.05).

Compared to Pod^TOM^ podocytes, *Myd88* deficient podocytes showed a similar expression profile during NTN, with only 125 significantly upregulated and 111 significantly downregulated genes (Fig. 5A, B). Pathway-based analysis of transcriptional changes indicated a subtle downregulation in the expression of genes important for integrity and function of tight junctions, including *Scrib, Synpo, Myh9, Actg1, Actn1* except for *Myl12a*, which did not follow this trend (Fig. 5C, D).

A more detailed analysis of chemokines and cytokines revealed a similar pattern of expression between injured MyD88^pko^ and Pod^TOM^ podocytes and their respective healthy controls, suggesting a compensatory mechanism for the *Myd88* deficiency. Analysis of TLR receptors and downstream signaling molecules revealed an increase in gene expression for TLR3 and transcription factor IRF3 along with IRF3 targets like *Ifi44* and *Ifit1* suggesting the presence of MYD88 independent signaling (Fig. 5E). In addition, glomerulonephritis caused the differential expression of various genes related to the type I interferon mediated signaling pathway, including an upregulation of *Irf3, Ifitm3*, *Ifnar2* and *Wnt5a* (Suppl. Figure 4).

## Discussion

Upon injury, podocytes undergo profound structural changes, including flattening of their foot processes, a phenomenon referred to as foot-process effacement. How podocytes sense external inflammatory stimuli is incompletely understood. In addition to their role as signal receivers, podocytes have the capacity to emit signals into their surroundings, thereby orchestrating the integrity of the glomerulus. One of the most prominent signaling molecules originating in podocytes is VEGF1, which plays a vital role in maintaining the health of glomerular endothelial cells^28^. In addition, podocytes are responsible for generating NF-κB-dependent pro-inflammatory signals, which actively contribute to the progression of murine crescentic glomerulonephritis^5^.

To understand the adaptive changes in the podocyte transcriptome during GN, we conducted bulk RNA sequencing of sorted podocytes from mice undergoing NTN and their healthy littermates. Injured podocytes displayed significant regulation of genes associated with the control of the actin cytoskeleton. The most enriched GO:terms were linked to the regulation of cell–cell interactions, including tight junctions and focal adhesions, as well as components of the cytoskeleton and signaling pathways involving Rho GTPases. These results highlight the significance of dynamic changes in podocyte structure during the diseased state. Among the genes that were notably upregulated, we observed the actin bundling molecule synaptopodin, as well as ARHGAP23 and tropomyosin 1 presumably representing an attempt to stabilize the podocyte actin cytoskeleton. Synaptopodin and tropomyosin were found to work in concert to presumably reinforce RhoA activity^29,30^. ARHGAP23, a relatively lesser-known Rho-GAP, was recently discovered to stabilize neuronal synapses by deactivating RAC1^31^. While the role of ARHGAP23 in podocytes remains unclear, its substantial upregulation suggests potential importance, especially considering the impact of other ARHGAPs like ARHGAP24 on the podocyte cytoskeleton^32^. Mice expressing constitutively active RAC1 develop rapid onset proteinuria, foot process effacement, and increased motility^33,34^.

As expected, several genes encoding for pro-inflammatory chemokines, such as *Cxcl1*, were significantly upregulated in the inflamed condition. CXCL1 is an NF-κB-dependent potent neutrophil chemoattractant that was found to be expressed in *Actn4* KO podocytes and upregulated in FSGS kidney biopsies^35^. Among the most downregulated genes, the E3-ubiquitin ligase NEDD4 was recently found to be involved in ubiquitin-mediated proteasomal degradation of nephrin^36^. Therefore, this downregulation might represent an attempt to stabilize nephrin under stress.

As TLRs are among the most important sensors of an inflammatory environment, it was unexpected that a knockout of the central adaptor protein MYD88 in podocytes did not result in a significant alteration of experimental GN, especially as a podocyte-specific knockout of the NF-κB adaptor NEMO resulted in a significant attenuation of GN^5^. There are two plausible explanations for this observation. On the one hand, reducing podocyte-intrinsic MYD88-dependent TLR signaling alone may have minimal impact on the highly inflammatory environment during NTN, which is primarily orchestrated by endothelial cells and leukocytes. On the other hand, it is possible that the existence of MYD88-independent signaling pathways upstream of the NEMO/Ikkα/Ikkβ complex compensate for the loss of MYD88. In this context, TLR3 and TLR4 signaling may be important, as both can activate NF-κB signaling and Type I interferon responses via IRF3 independently of MYD88^8,9^. Indeed, we observed an induction of *Irf3* and its downstream targets, *Ifi44* and *Ifit1*, with a significant differential expression of *Isg15* when comparing nephritic and healthy MyD88^pko^ mice. While our study suggests the potential involvement if IRF3-related signaling in podocyte injury, this finding has to be confirmed experimentally in future studies. The identification and a better understanding of these compensatory processes is important for our understanding of the role of podocytes in the pathophysiology of inflammatory glomerular diseases and for the development of more specific therapeutic strategies. The recently published *Irf3*^flox^ mouse now offers the opportunity to investigate the role of this molecule and interferon-related signaling specifically in podocytes in detail^37^.

Another limitation of our study is the use of a single disease model, as MYD88 might have context-dependent functions in podocytes. While our model causes glomerular injury by the deposition of immune complexes, the cytotoxic adriamycin model might be more suitable to analyze the impact of MyD88 on podocyte survival in future studies.

In summary, our study is the first to inhibit MYD88-dependent TLR signaling specifically in podocytes. In addition to shedding light on the adaptive mechanisms involved in immune-complex-mediated podocyte injury, this study highlights the significance of MYD88-independent signaling pathways in recognizing and responding to cellular injury. This prompts the question of whether these pathways might be potential therapeutic targets in the future.

### Supplementary Information

Supplementary Figures.

## Data Availability

Bulk RNA-seq data is available at Gene Expression Omnibus (GEO) with accession number GSE222625. Other data underlying this article is either available in the article and its supplementary material or will be shared on a reasonable request to the corresponding author.
