# Supplementary material for: The podocytes’ inflammatory responses in experimental GN are independent of canonical MYD88-dependent toll-like receptor signaling
Source: Sci Rep. 2024 Jan 27;14:2292. doi: 10.1038/s41598-024-52565-8 (PMC10821883; doi:10.1038/s41598-024-52565-8)
Supplement: Supplementary file 1 — Supplementary Figures. [file 41598_2024_52565_MOESM1_ESM.pdf]

**Data supplement:**

**Supplemental Figure 1. Podocyte specific *Myd88* knock out does not alter kidney function in 52 weeks old mice**
**(A)** No significant difference is detected in urine albumin-to-creatinine-ratio (ACR) and blood urea nitrogen (BUN) in 52 weeks old, untreated $MyD88^{pko}$ (n=5) and WT littermates (n=5).

**Supplemental Figure 2. Semi-automated analysis of the podocyte ultrastructure does not reveal significant differences between $MyD88^{pko}$ and WT mice during NTN**
**(A)** Representative pictures of sheep IgG (magenta) and podocin (green) co-stainings show glomerular nephrotoxic IgG deposition at day 10 of NTN. No difference was observed between the groups. **(B)** Representative, pre-adjusted STED images and semi-automated segmentation of the slit diaphragm (red) at indicated time point after NTN induction. The cyan line indicates the ROI in which the analysis was carried out. The slit diaphragm length is used to compare the extend of podocyte damage. No significant difference was observed in $MyD88^{pko}$ mice compared to WT littermates.

**Supplemental Figure 3. Sorting of podocytes before bulk RNA sequencing**
**(A)** Urine albumin-to-creatinine-ratio (ACR) of nephritic $MyD88^{pko}$ and control mice before extraction of mTomato-positive podocytes using FACS. (**B**) Exemplary gating strategy of mTomato-positive podocytes. No significant difference was observed in purity and viability of FACS sorted podocytes before bulk RNA sequencing.

**Supplemental Figure 4. Differential expression of type-I interferon related genes in experimental GN.**
Expression profiles of differentially expressed genes associated with the type-I interferon signaling pathway in sorted podocytes between indicated groups (p-value<0.05). The bulk RNA-seq data was searched for genes related to the Gene Ontology term GO: 0060337. If multiple isoforms were present, only the one with CCDS entry, high transcript support level and highest effect size (in that order) was considered.

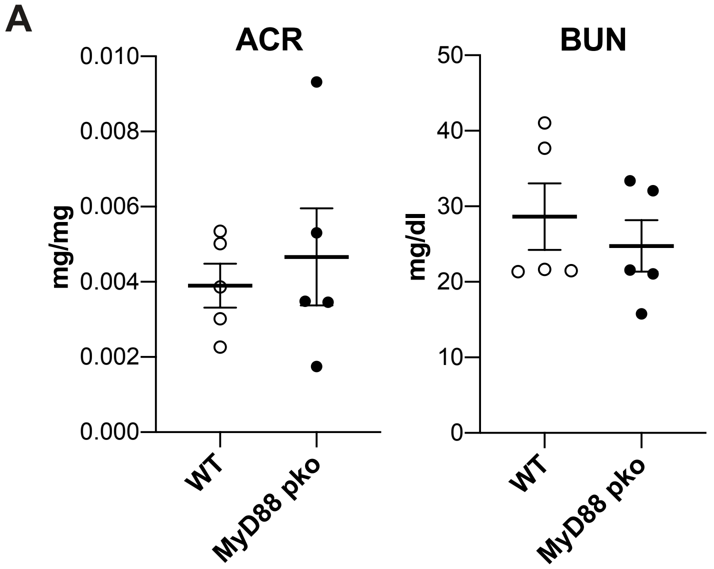


**Supplemental Figure 1. Podocyte specific *Myd88* knock out does not alter kidney function in 52 weeks old mice**

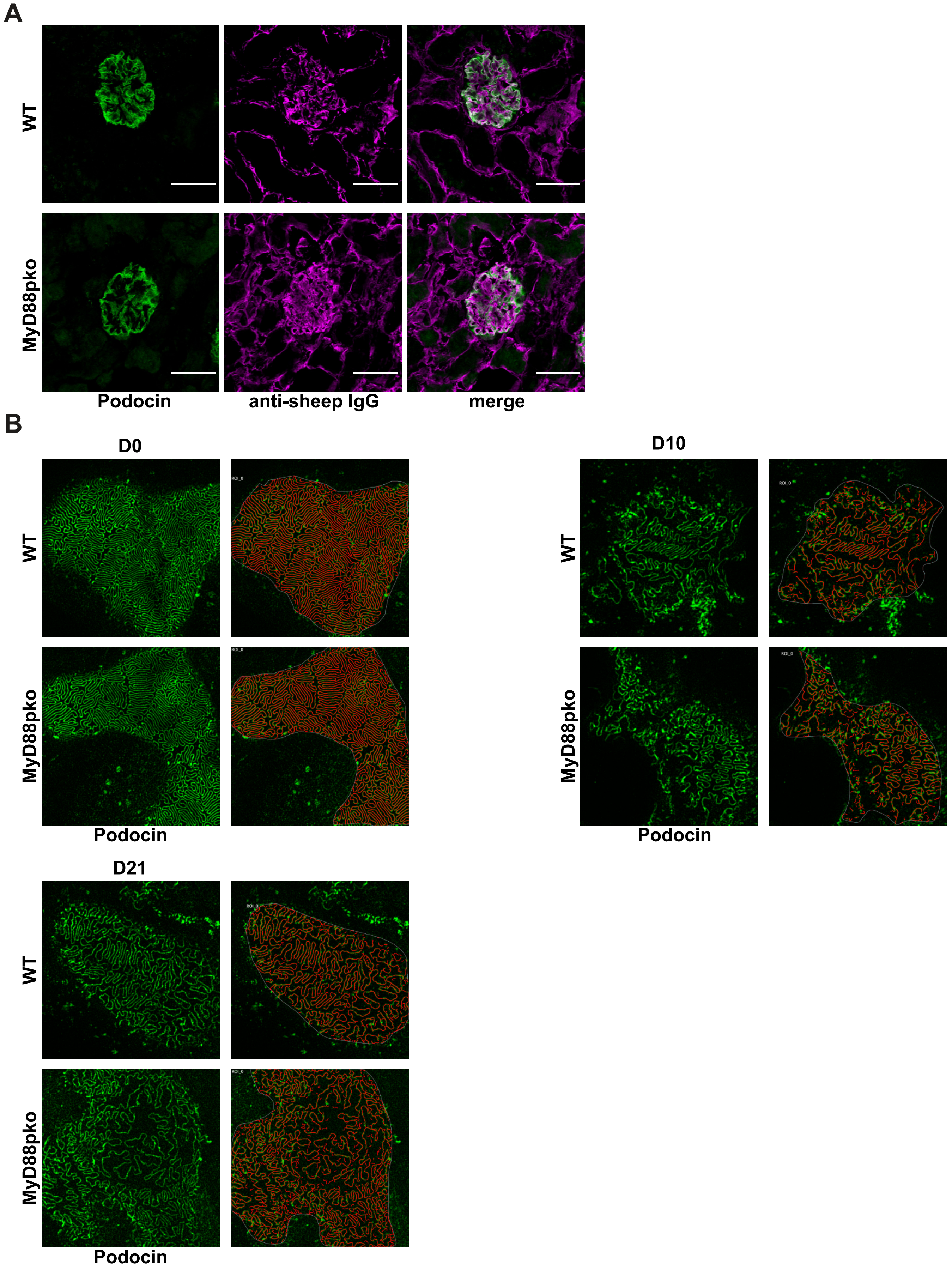


**Supplemental Figure 2. Semi-automated analysis of the podocyte ultrastructure does not reveal significant differences between MyD88$^{pko}$ and WT mice during NTN**

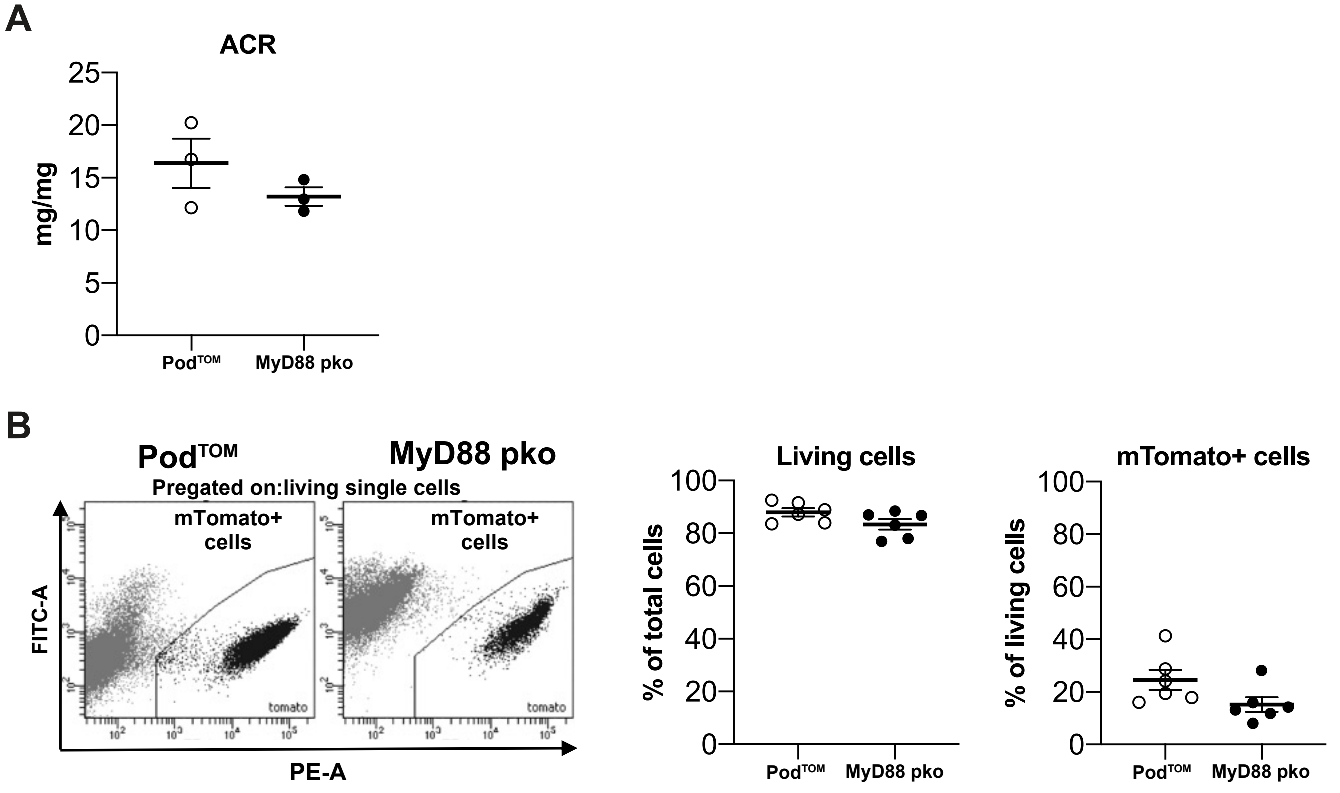


**Supplemental Figure 3. Sorting of podocytes before bulk RNA sequencing**

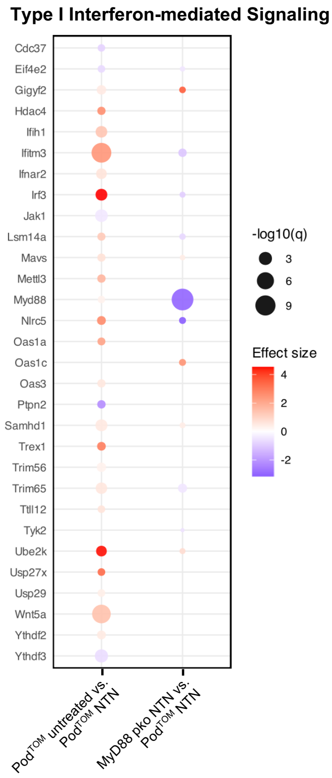


**Supplemental Figure 4. Differential expression of type-I Interferon related genes in experimental GN.**
